# Heart disease detection based on internet of things data using linear quadratic discriminant analysis and a deep graph convolutional neural network

**DOI:** 10.3389/fncom.2022.964686

**Published:** 2022-10-07

**Authors:** K. Saikumar, V. Rajesh, Gautam Srivastava, Jerry Chun-Wei Lin

**Affiliations:** ^1^Department of ECE, Koneru Lakshmaiah Education Foundation, Green Fields, Vaddeswaram, Andhra Pradesh, India; ^2^Department of Mathematics and Computer Science, Brandon University, Brandon, MB, Canada; ^3^Research Centre for Interneural Computing, China Medical University, Taichung, Taiwan; ^4^Department of Mathematics and Computer Science, Lebanese American University, Beirut, Lebanon; ^5^Western Norway University of Applied Science, Bergen, Norway

**Keywords:** heart disease, detection, IoT - internet of things, sensor data, deep learning, artificial neural network

## Abstract

Heart disease is an emerging health issue in the medical field, according to WHO every year around 10 billion people are affected with heart abnormalities. Arteries in the heart generate oxygenated blood to all body parts, however sometimes blood vessels become clogged or restrained due to cardiac issues. Past heart diagnosis applications are outdated and suffer from poor performance. Therefore, an intelligent heart disease diagnosis application design is required. In this research work, internet of things (IoT) sensor data with a deep learning-based heart diagnosis application is designed. The heart disease IoT sensor data is collected from the University of California Irvine machine learning repository free open-source dataset which is useful for training the deep graph convolutional network (DG_ConvoNet) deep learning network. The testing data has been collected from the Cleveland Clinic Foundation; it is a collection of 350 real-time clinical instances from heart patients through IoT sensors. The K-means technique is employed to remove noise in sensor data and clustered the unstructured data. The features are extracted to employ Linear Quadratic Discriminant Analysis. DG_ConvoNet is a deep learning process to classify and predict heart diseases. The diagnostic application achieves an accuracy of 96%, sensitivity of 80%, specificity of 73%, precision of 90%, F-Score of 79%, and area under the ROC curve of 75% implementing the proposed model.

## Introduction

According to WHO, cardiovascular disease (CVD) is a significant reason of death worldwide, with 17.8 million deaths every decade (Rath et al., 2021). The American Cardiac Organization (Zhang and Xu, 2021) specifies detailed indications like sleep disorders, slight pain increase as well as a drop-in heart rate and fast weight improvement (up to 1.5-2.5 kg per 7 days) (Vincent Paul et al., 2021). However, more study data and patient records from hospitals become available as time goes on. Machine learning (ML) and artificial intelligence (AI) are now widely recognized as able to play a vital role in the medical industry. ML and deep learning (DL) methods are often used to diagnose conditions as well as classify or anticipate results. ML algorithms can do a complete examination of genetic data in a short amount of time. Medical records are modified and analyzed extra thoroughly for improved predictions, and methods are trained for knowledge pandemic predictions (Liu et al., 2022). Heart disorders are identified with congenital, coronary and rheumatic events, and 370,000 Americans died due to coronary heart disease (HD) type heart attacks in 2015. Annually Americans are spending $250 billion USD on HD diagnosis and treatment. According to the American heart association, medical HD disorders will be able to be predicted by 2030.

Exercise stress tests, chest X-rays, CT scans, MRI, coronary angiograms, and electrocardiograms (ECG) are currently used to diagnose the severity of HD in patients. Patients need early and precise diagnoses of coronary HD to receive timely and effective treatment and boost their chances of long-term survival. Unfortunately, cardiovascular specialists may not be available in many resource-limited places worldwide to do these diagnostic tests. Missing diagnoses, incorrect diagnoses, and therapies put patients’ health in danger in many circumstances. In addition, early detection of HD causes preventative interventions such as drugs, lifestyle changes, angioplasty, or surgery, which can help to slow disease development as well as minimize morbidity (Morris and Lopez, 2021). As a result, precise and timely heart disease diagnostics are critical for lowering mortality as well as enhancing long-term survival rates in patients. Because early detection of coronary HD is challenging, computer-assisted techniques for detecting and diagnosing heart disease in people have been developed. In medical institutions, ML methods that analyze clinical data, evaluate it, and diagnoses medical conditions is becoming increasingly common in healthcare fields.

The research contributions of this paper are as follows:

1. Collect internet of things (IoT) sensor-based heart disease data in the detection of heart disease using a deep learning architecture.
2. Process input data for noise removal and cluster the data using K-means clustering.
3. Extract the features using Linear Quadratic Discriminant Analysis.
4. Classify the extracted data using a deep graph convolutional network (DG_ConvoNet).

### Appendix

Internet of things (IoT), World Health Organizations (WHO), cardiovascular disease (CVD), Receiver Operating Characteristic Curve (ROC), Machine learning (ML), Artificial Intelligence (AI), Deep learning (DL), Heart-Disease (HD), Support Vector Machine (SVM), Heart Rate Variability (HRV), Convolution Neural Network (CNN), Magnetic Resonance Imaging (MRI), Deep Graph Convolutional Network (DG_ConvoNet).

### Related work

In this section, a brief literature has been employed from the latest research papers related to heart disease prediction using IoT sensor data. Feature extraction, classification and predictions are the major steps involved in intelligence algorithms. Manogaran et al. (2017) utilized a variety of big data methods to detect cardiac illness, as well as hyperparameter tuning to improve the accuracy of results. Kanksha Aman et al. (2021) employed generalized discriminant analysis for extracting nonlinear HD features. A binary classifier with extreme ML has been used to reduce overfitting issues as well as increase training time on finding heart disorders prediction. For detecting coronary HD, the accuracy was 73% had been attained which was very less. Heart rate variability was classified as an arrhythmia by Divya et al. (2021). The heart abnormality disorders classification was done with a multilayer perceptron neural network, and 91% accuracy was reached by decreasing features or using Gaussian Discriminant Analysis, in this research work hidden features haven’t been included. Hasan and Bhattacharjee (2019) employed Gaussian discriminant analysis to reduce HRV signal characteristics to 15 and an SVM classifier to obtain 70% precision, this research work cannot solve unstructured sensor data from IoT networks. An enhanced CNN model is proposed by Huang et al. (2019), in which 92.35% accuracy had been detected, the main limitation of this work is STFT-based spectrogram analysis. The STFT model is very old and faces clustering issues when large datasets have been applied to it. The Fruit classification is a complex process to predict heart diseases through IoT sensor data. The following challenge was solved by using a CNN-based technique by Wang et al. (2020). According to the researchers, designed past HD detection methods has a less classification accuracy, which is get improved than the existing methods. Zhang et al. (2019) proposed a comprehensive description of multimodal data fusion of heart-related sensor data. A combination of CT, MRI, PET, optical imaging and radionuclide datasets has resulted in complete pathology of heart disorders in a radiology manner. The image fusion-based approach has been found to improve clinical diagnosis in recent years but failed at emergency diagnosis conditions. The CNN-based diagnosis algorithm implemented by Zhang et al. (2020a). In this research, stochastic pooling, as well as optimization of hyperparameters connected with CNN. The major drawback of this study is neuroimages orientation is altered from patient to patient so that when applying a new image to the designed application, the HD abnormality detection rate had been getting changes extremely.

The realized methods which are shown in Table 1 have less operational sensitivity, specificity, and accuracy. Zhang et al. (2020b) introduced an FGCNet-based HD features extraction from GCN and CNN models. This method is used to diagnose chest CT scan-based heart disorders prediction but fails at noise-based CT scan radiology images specified as test input. The FGCNet is said to aid quick COVID 19 detection utilizing chest CT scans. Wang et al. (2021a) presented the CCSHNet method for heart disorders detection, which combines deep fusion. The designed CCSHNet models failed at large data samples applied at the training stage. The DCA and transfer learning-based models are very critical to detecting HD at large dimensional data. The CCSHNet is a viable option for detecting infectious heart illnesses, including COVID 19, according to deep exhaustive analysis. The literature review from many latest articles identified that traditional ML-based detection of arrhythmia with ECG signals analysis methods are outdated. However, fewer research works have been published on HD detection utilizing ECG signals and DL techniques are trending but IoT-related works are not much efficient to predict HD. Wang et al. (2021b) evaluate classification algorithms using an ML technique to predict cardiac disease. This work demonstrated the bagging technique prediction for HD with a good performance rate, as well as accuracy level. Superior HD prediction models other than past techniques are necessary. Martins et al. (2021) offer a genetic approach for predicting human heart disease through echocardiographic, the designed method is limited to huge unstructured data. The implemented method might reduce the number of test cases required to detect HD issues based on Ali et al. (2021) and Ladefoged et al. (2021). The successful HD abnormality prediction based on the radiology dataset is outdated as well as latest IoT-based techniques are required. Saikumar et al. (2022) aim to develop a precise categorization algorithm for accurately predicting cardiac disease but are unable to work on IoT sensor data. The following work concluded that regression classification is used to predict HD more accurately than other techniques by Saikumar and Rajesh, 2020a,b. R-C4.5 is proposed, and its features are extractí from the given technique by Koppula et al. (2021). The study used their equipment and found it a very beneficial machine in the healthcare industry for predicting ML-based approaches Garigipati et al. (2022). The above discussions are providing information about earlier HD prediction models and its limitations. It is clear that many cardiac diagnosis models are facing various low-level and high-level issues under dynamic conditions. This research work looks to solve some of the indicated issues from the related works.

**TABLE 1:** Recent studies related to heart abnormality prediction.

S No	Author	Techniques	Performance accuracy	Limitation
1	Rahmani et al., 2018	IoT based e-health	Accuracy = 93.24% HD Detection rate = 0.76 Application score = 0.86	Data clustering is a complex issue
2	Majumder et al., 2019	Smart IoT-based cardiac disorders detection	Accuracy = 95.23% HD Detection rate = 0.79 Application score = 0.72	Limited large dimensional data
3	Golande et al., 2019	Smart medical applications using IoT	Accuracy = 91.47% HD Detection rate = 0.86 Application score = 0.83	Network issue due to conventional data analysis
4	Haq et al., 2018	ML-based HD detection	Accuracy = 95.42% HD Detection rate = 0.83 Application score = 0.71	Radiology data-based analysis is sometimes altered from sample to sample
5	Hinton and Salakhutdinov, 2006	High dimensional HD data-based abnormality detection	Accuracy = 93.68% HD Detection rate = 0.69 Application score = 0.82	Limited to structured HD data

## System model

This section discusses the proposed DL technique based on feature extraction as well as classification in heart disease diagnosis. Here, the input data has been collected as IoT sensor data from a patient monitoring system.

**FIGURE 1:**
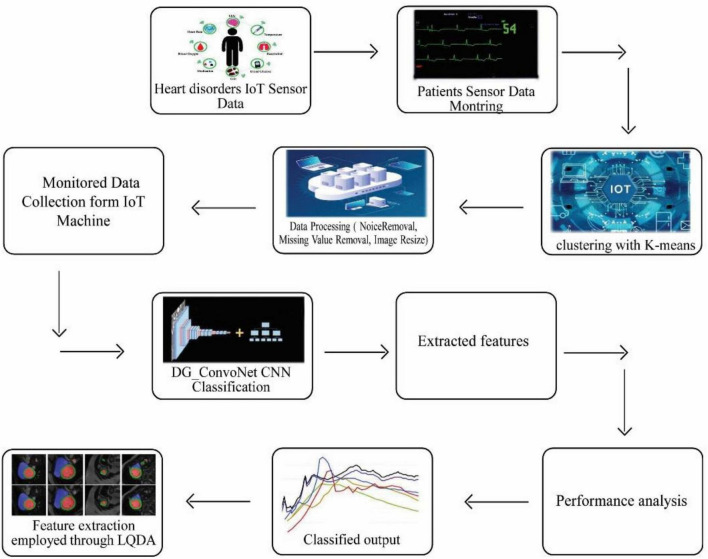
Proposed IoT sensor data-based heart disease (HD) prediction.

The collected data has been processed for noise abstraction using a clustered-based K-means algorithm. Gaussian noise that was present in the medical images was removed at this block. Clustered information is used to extract the features utilizing Linear Quadratic Discriminant Analysis. Finally, the extracted features have been classified using the DG_ConvoNet. The architecture of the proposed method is shown in Figure 1. The pre-processing unit categorizes image registration from the medical raw image data (University of California Irvine machine learning repository). The registration enhancement process is used to line up the image for de-noise processing. Due to speckle disturbances, medical images get damaged and hinder the ability to identify deep features needed for DL. As a result, medical images are de-specked using a filtering approach technique to improve categorization results.

### K-means clustering

Since k represents the number of clusters, there are k centroids, one for every cluster. After the Euclidean distance between each data point and the centroid has been evaluated, the assignment of data points to the centroid is based on the shortest Euclidean distance from that centroid. An early grouping is done when no point is left unassigned. Now, k new centroids are generated, and the iteration continues until the k centroids’ positions do not change. In this stage, 256 clusters had to be created and processed for the centroid calculation of the cluster.

Let Y = {x_1_, x_2_, x_3_, …, …, x_n_} are set of dataset opinions as well as Z = {z1, z2, …, …z_c_} be set of centers.

1. Arbitrarily choose ‘c’ cluster centers.
2. Evaluate the distance among each information point as well as cluster centers.
3. Allot data points to the cluster center with the shortest distance between it and all other cluster centers.
4. Again, evaluate the original cluster center using the following Eq. (1):

(1) $${\text{Z}}_{\text{i}}=\left(1/{\text{c}}_{\text{i}}\right).\Sigma_{\text{j}=1}^{{𝔼}_{1}}\,{\text{x}}_{𝕀}$$

5. Where ‘c_i_’ indicates the number of data opinions in the ith cluster.
6. Again, calculate the distance between every data point as well as the original cluster centers.
7. Stop if no information points were reallocated; otherwise, start over at step 3.

**FIGURE 2:**
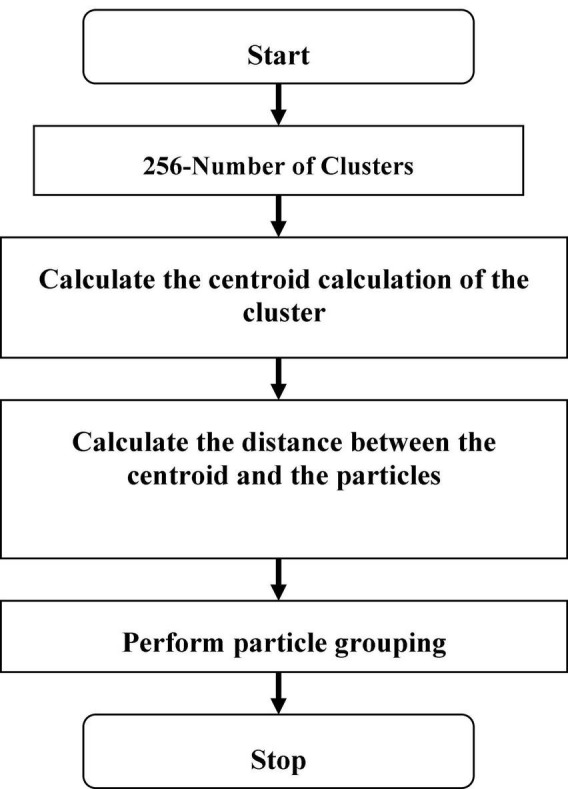
Flow chart K-means.

The flow chart of K-means clustering is shown in Figure 2. In this K-means flow is explained with clustered extraction on the dataset. The centroid, Euclidean and particle estimation parameters have been providing information about deep dataset information. The dataset consists of shape-based image features which are processed by the K-means algorithm.

### Linear quadratic discriminant analysis based feature extraction

Let S_b_ and S_w_ be among and within-class scatter matrices, low-dimensional complement space of null space of S_b_, related as 𝒮′, is first extracted. Let V_b_ = [v_b1_, …, v_bM_] be M eigenvectors of S_b_ corresponding to M non-zero eigenvalues A = [λ_b1_, …, λ_b_M], where M = min(C−1, J). The S_b_ subspace ℬ′ is thus spanned by V_b_, which is further scaled by $\text{U}={\text{V}}_{\text{b}}\,{\text{A}}_{\text{b}}^{-1/2}$ so that U_T_S_b_U = ℐ, where A_b_ = diag (A), diag()indicates the diagonalization operator and ℐ is the (M = M) identity matrix by Eq. (2):


$${\overset{`}{\Sigma }}_{\text{i}}\,\left(\alpha ,\gamma \right)=\left(1-\gamma \right)\,{\overset{`}{\Sigma }}_{\text{i}}\,\left(\alpha \right)+\frac{\gamma }{\text{M}}\,\text{tr}\,\left[{\overset{`}{\Sigma }}_{\text{i}}\,\left(\alpha \right)\right]\,\text{I},$$

(2) $${\overset{`}{\Sigma }}_{\text{i}}\,\left(\alpha \right)=\frac{1}{{\text{C}}_{\text{i}}\,\left(\alpha \right)}\,\left[\left(1-\alpha \right)\,{\text{S}}_{\text{i}}+\alpha \,\text{S}\right],$$

M is the dimensionality of *ℬ*′.C_i_(α) = (1−α)C_i_ + αN and S_i_ is the covariance matrix of ith class evaluated in *ℬ*′, i.e., ${\text{S}}_{\text{i}}=\Sigma_{\text{j}=1}^{{\text{C}}_{\text{i}}}\,\left({\text{y}}_{\text{ij}}-{\bar{\text{y}}}_{\text{i}}\right)\,{\left({\text{y}}_{\text{ij}}-{\bar{\text{y}}}_{\text{i}}\right)}^{\text{T}},{\text{y}}_{\text{ij}}={\text{U}}^{\text{T}}\,{\text{z}}_{\text{ij}},{\bar{\text{y}}}_{\text{i}}=\left(1/{\text{C}}_{\text{i}}\right)\,\,\Sigma_{\text{j}=1}^{{\text{C}}_{\text{i}}}\,{\text{y}}_{\text{ij}}$ and $\text{S}=\Sigma_{\text{i}=1}^{\text{C}}\,{\text{S}}_{\text{i}}.$

Let Φ = [ϕ(z_11_), …, ϕ(z_CCc_)] be corresponding feature representations of training samples in kernel space 𝔽^F^. Let K be N = N Gram matrix, i.e., $\text{K}={\left({\text{K}}_{\text{lh}}\right)}_{\text{l = l,,C}}^{\text{h }=\;l,,{\text{C}}_{\text{Ih}}}$ is a C_1_ × C_h_ sub - matrix of K composed of samples from classes ℐ_l_ and 𝒵_h_, i.e., ${\text{K}}_{\text{lh}}={\left({\text{k}}_{\text{ij}}\right)}_{\text{i}=1,.,{\text{C}}_{\text{l}}}^{\text{j}=1,}$, where k_ij_ = k(z_li_,z_hj_) and k(⋅)indicates kernel function defined in ℝ^J^. Let ${\bar{\text{S}}}_{\text{b}}$ be between-class scatter in 𝔽^F^, described as Eq. (3)


(3) $${\overset{`}{\text{S}}}_{\text{b}}\;=\;\frac{1}{\text{N}}\overset{\text{C}}{\underset{\text{i}\;=1}{\Sigma }}{\text{C}}_{\text{i}}\left({\overset{↼}{\phi }}_{\text{i}}-\overset{↼}{\phi }\right){\left({\overset{↼}{\phi }}_{\text{i}}-\overset{↼}{\phi }\right)}^{\text{T}}$$

where ${\overset{↼}{\phi }}_{\text{i}}=(1/{\text{C}}_{\text{i}}){\text{Σ}}_{\text{j}=1}^{{\text{C}}_{\text{i}}}\phi ({\text{z}}_{\text{ij}})$ is the mean of 𝒴_i_ in 𝔽F and $\overset{↼}{\phi }=\left(1/N\right)\,\Sigma_{\text{i}=1}^{\text{C}}\,\Sigma_{\text{j}=1}^{{\text{C}}_{\text{i}}}\,\phi \,\left({\text{z}}_{\text{ij}}\right)$ is mean of training samples FF.

Eigenvectors of S_b_, i.e., ${\overset{`}{\text{V}}}_{\text{b}}=\left[{\bar{\text{v}}}_{\mathrm{b1}},\ldots ,{\bar{\text{v}}}_{\text{bM}}\right]$, corresponding to M largest eigenvalues. ${\overset{`}{\text{V}}}_{\text{b}}$ is obtained by solving the eigenvalue issue of ${\bar{\text{S}}}_{\text{b}}$, which is represented as Eq. (4):


$${\bar{s}}_{b}={\sum_{i=1}^{\text{c}}\left(\sqrt{\frac{ci}{N}}({\overset{↼}{\phi }}_{i}-\overset{↼}{\phi })\right)\left(\sqrt{\frac{ci}{N}}({\overset{↼}{\phi }}_{i}-\overset{↼}{\phi })\right)}^{T}$$

(4) =∑i=1cϕ↼`ϕ↼`i=iTΦbΦbT

where ${\overset{`}{\phi }}_{\text{i}}=\sqrt{{\text{C}}_{\text{i}}/\text{N}}\left({\overset{↼}{\phi }}_{i}-\overset{↼}{\phi }\right)$ and $\Phi_{\text{b}}=\left[{\overset{`}{\phi }}_{1},\ldots ,{\overset{`}{\phi }}_{\text{C}}\right]$. It is given that ${\bar{\text{S}}}_{\text{b}}$ is a matrix of size F × F, where F indicates kernel space dimensionality. Due to HD of 𝔽^F^, a direct computation of eigenvectors of ${\bar{\text{S}}}_{\text{b}}$ is impossible$\left(\Phi_{\text{b}}\,\Phi_{\text{b}}^{\text{T}}\right)\,\left(\Phi_{\text{b}}\,{\bar{\text{e}}}_{\text{bi}}\right)=\overset{↼}{\lambda }\left(\Phi_{\text{b}}\,{\bar{\text{e}}}_{\text{bi}}\right)$. Therefore, it is deduced that $\left(\Phi_{\text{b}}\,{\bar{\text{e}}}_{\text{bi}}\right)$ is the i th eigenvector of ${\bar{\text{S}}}_{\text{b}}=\Phi_{\text{b}}\,\Phi_{{\text{b}}^{-}}^{\text{T}}$


$$\Phi_{b}^{T}\Phi_{b}=\frac{1}{N}B(A_{\mathrm{NC}}^{T}\cdot K\cdot A_{\mathrm{NC}}-\frac{1}{N}\left(A_{\mathrm{NC}}^{T}\cdot K\cdot 1_{\mathrm{NC}}\right)$$

(5) $$-\frac{1}{N}\left(1_{\mathrm{NC}}^{T}\cdot K\cdot A_{\mathrm{NC}}\right)+\frac{1}{N^{2}}\left(1_{\mathrm{NC}}^{T}\cdot K\cdot 1_{\mathrm{NC}}\right))B$$

where $\text{B}=\text{diag}\,\left[\sqrt{{\text{C}}_{1}},\ldots ,\sqrt{{\text{C}}_{\text{C}}}\right],1_{\text{NC}}$ is an N × C matrix with all elements equal to 1,A_NC_ = diag[a_C_1__,…,a_C_C__] is an N × C block diagonal matrix and a C_i_ is a C_i_ = 1 vector with all elements equal to 1/C_i_. Let ${\bar{\text{E}}}_{\text{bM}}=\left[{\bar{\text{e}}}_{\mathrm{b1}},\ldots ,{\overset{`}{\text{e}}}_{\text{bM}}\right]$ consist of M significant eigenvectors of $\Phi_{\text{b}}^{\text{T}}\,\Phi_{\text{b}}$ corresponding to M largest eigenvalues ${\overset{↼}{\lambda }}_{\text{b1}}>,...,>{\overset{↼}{\lambda }}_{\text{b}}\text{M}$ and ${\overset{`}{\text{V}}}_{\text{b}}=\Phi_{\text{b}}\,{\bar{\text{E}}}_{\text{bM}}$, it is not difficult to derive that ${\bar{\text{V}}}_{\text{b}}^{\text{T}}\,{\bar{\text{S}}}_{\text{b}}\,{\bar{\text{V}}}_{\text{b}}=\overset{↼}{\Lambda }$, where ${\overset{↼}{\wedge }}_{\text{b}}=\text{diag}\left[{\overset{↼}{\lambda }}_{\text{b},1,...,}^{2}{\overset{↼}{\lambda }}_{\text{b,M}}^{2}\right]$. Thus, the transformation matrix $\vec{\text{U}}$ such that ${\bar{\text{U}}}^{\text{T}}\,{\overset{`}{\text{S}}}_{\text{b}}\,\bar{\text{U}}=\text{I}$is evaluated as Eqs. (6), (7):


(6) $$\bar{\text{U}}=\bar{{\text{V}}_{\text{b}}}{\overset{↼}{\text{A}}}_{\text{b}}^{-1/2},\bar{{\text{V}}_{\text{b}}}={\text{Φ}}_{\text{b}}{\overset{`}{\text{E}}}_{\text{b}\text{M}}$$

(7) $${\overset{`}{\text{y}}}_{\text{ij}}={\bar{\text{U}}}^{\text{T}}\phi ({\text{z}}_{\text{ij}})={\overset{↼}{\text{A}}}_{\text{b}}^{-1/2}{\bar{\text{E}}}_{\text{bM}}^{\text{T}}\Phi_{\text{b}}^{\text{T}}\phi ({\text{z}}_{\text{ij}})$$

where $\Phi_{\text{b}}^{\text{T}}\,\phi \,\left({\text{z}}_{\text{ij}}\right)$ can be expressed as Eq. (8)


(8) $${\text{Φ}}_{\text{b}}^{\text{T}}\,\phi \,\left({\text{z}}_{\mathrm{ij}}\right)=\frac{1}{\sqrt{\text{N}}}\,\text{B}\,\left({\text{A}}_{\text{NC}}\cdot \text{v}\,\left(\phi \,\left({\text{z}}_{\mathrm{ij}}\right)\right)-\frac{1}{\text{N}}\,1_{\text{NC}}^{\text{T}}\cdot \text{v}\,\left(\phi \,\left({\text{z}}_{\mathrm{ij}}\right)\right)\right)$$

where v(ϕ(z_ij_)) = [ϕ(z_11_)ϕ(z_ij_),ϕ(z_12_)ϕ(z_ij_),…,ϕ(z_CC_C__)ϕ(z_ij_)]^T^ is evaluated implicitly through the kernel function described in ℝ^J^, i.e., ϕ(z_mn_)ϕ(z_ij_) = k(z_mn_, z_ij_).


$${\overset{`}{\Sigma }}_{\text{i}}\,\left(\alpha ,\gamma \right)=\left(1-\gamma \right)\,{\overset{`}{\Sigma }}_{\text{i}}\,\left(\alpha \right)+\frac{\gamma }{\text{M}}\,\text{tr}\,\left[{\bar{\Sigma }}_{\text{i}}\,\left(\alpha \right)\right]\,\text{I},$$

$${\overset{`}{\Sigma }}_{\text{i}}\,\left(\alpha \right)=\frac{1}{{\text{C}}_{\text{i}}\,\left(\alpha \right)}\,\left[\left(1-\alpha \right)\,{\bar{\text{S}}}_{\text{i}}+\alpha \,\bar{\text{S}}\right],$$

$${\text{C}}_{\text{i}}\,\left(\alpha \right)=\left(1-\alpha \right)\,{\text{C}}_{\text{i}}+\alpha \,\text{N},$$

$${\overset{`}{\text{S}}}_{\text{i}}=\sum_{\text{j}=1}^{{\text{C}}_{\text{i}}}\left({\bar{\text{y}}}_{\mathrm{ij}}-{\bar{\bar{\text{y}}}}_{\text{i}}\right)\,{\left({\bar{\text{y}}}_{\mathrm{ij}}-{\bar{\bar{\text{y}}}}_{\text{i}}\right)}^{\text{T}},$$

$$\bar{\text{S}}=\sum_{\text{i}=1}^{\text{C}}{\overset{`}{\text{S}}}_{\text{i}}.$$

(9) $${\bar{\bar{\text{y}}}}_{\text{i}}=\left(1/{\text{C}}_{\text{i}}\right)\,\sum_{\text{j}=1}^{{\text{C}}_{\text{i}}}{\bar{\text{y}}}_{\mathrm{ij}}$$

and (**α,γ**) is a pair of regularization parameters.

The key component in the evaluation of ${\bar{\Sigma }}_{\text{i}}\,\left(\alpha ,\gamma \right)$ is to arise covariance matrix of ith class, i.e., ${\bar{\text{S}}}_{\text{i}}$ which is given as Eq. (10):


$${\overset{`}{\text{S}}}_{\text{i}}=\sum_{\text{j}\,1}^{{\text{C}}_{\text{i}}}\left({\overset{`}{\text{y}}}_{\mathrm{ij}}-{\bar{\bar{\text{y}}}}_{\text{i}}\right)\,{\left({\bar{\text{y}}}_{\mathrm{ij}}-{\bar{\bar{\text{y}}}}_{\text{i}}\right)}^{\text{T}}$$

$$=\sum_{\text{j}\,1}^{{\text{C}}_{\text{i}}}{\bar{\text{y}}}_{\mathrm{ij}}\,{\bar{\text{y}}}_{\mathrm{ij}}^{\text{T}}-\sum_{\text{j}\,1}^{{\text{C}}_{\text{i}}}{\bar{\bar{\text{y}}}}_{\text{i}}\,{\bar{\text{y}}}_{\mathrm{ij}}^{\text{T}}-\sum_{\text{j}\,1}^{{\text{C}}_{\text{i}}}{\bar{\text{y}}}_{\mathrm{ij}}\,{\bar{\bar{\text{y}}}}_{\text{i}}^{\text{T}}+\sum_{\text{j}\,1}^{{\text{C}}_{\text{i}}}{\bar{\overset{`}{\text{y}}}}_{\text{i}}^{\text{T}}\,{\bar{\bar{\text{y}}}}_{\text{i}}^{\text{T}}$$

$$=\sum_{\text{j}\,1}^{{\text{C}}_{\text{i}}}{\bar{\text{y}}}_{\mathrm{ij}}\,{\overset{`}{\text{y}}}_{\mathrm{ij}}^{\text{T}}-{\text{C}}_{\text{i}}\,{\bar{\bar{\text{y}}}}_{\text{i}}\,{\bar{\overset{↼}{\text{y}}}}_{\text{i}}^{\text{T}}-{\text{C}}_{\text{i}}\,{\bar{\bar{\text{y}}}}_{\text{i}}\,{\bar{\bar{\text{y}}}}_{\text{i}}^{\text{T}}+{\text{C}}_{\text{i}}\,{\bar{\bar{\text{y}}}}_{\text{i}}\,{\bar{\bar{\text{y}}}}_{\text{i}}^{\text{T}}$$

$$=\sum_{\text{j}\,1}^{{\text{C}}_{\text{i}}}{\overset{`}{\text{y}}}_{\mathrm{ij}}\,{\bar{\text{y}}}_{\mathrm{ij}}^{\text{T}}-{\text{C}}_{\text{i}}\,{\bar{\bar{\text{y}}}}_{\text{i}}\,{\bar{\bar{\text{y}}}}_{\text{i}}^{\text{T}}$$

(10) $$={\text{J}}_{1}-{\text{C}}_{\text{i}}\times {\text{J}}_{2},$$

where ${\text{J}}_{1}=\Sigma_{\text{j}=1}^{{\text{C}}_{\text{i}}}\,{\overset{`}{\text{y}}}_{\text{ij}}\,{\bar{\text{y}}}_{\text{ij}}^{\text{T}}$ and ${\text{J}}_{2}={\bar{\bar{\text{y}}}}_{\text{i}}\,{\bar{\bar{\text{y}}}}_{\text{i}}^{\text{T}}$. The detailed derivation of J_1_ and J_2_ is determined in Appendices A and B.

Mahalanob is distance between feature representation of test image $\bar{\text{q}}$ and each class centre ${\bar{\bar{\text{y}}}}_{\text{i}}$ is then used to identify the test image. i.e., $\text{ID}\,\left(\text{p}\right)=\arg{\text{min}}_{\text{i}}\,{\text{d}}_{\text{i}}\,\left(\bar{\text{q}}\right)$, that can be calculated in Eq. (11) as:


$${\text{d}}_{\text{i}}\,\left(\bar{\text{q}}\right)={\left(\bar{\text{q}}-{\bar{\bar{\text{y}}}}_{\text{i}}\right)}^{\text{T}}\,{\overset{`}{\Sigma }}_{\text{i}}^{-1}\,\left(\alpha ,\gamma \right)\,\left(\bar{\text{q}}-{\bar{\bar{\text{y}}}}_{\text{i}}\right)+\text{ln}\,\left|{\bar{\Sigma }}_{\text{i}}\,\left(\alpha ,\gamma \right)\right|-2\,l\,n\,\pi_{\text{i}},$$

where π_i_ = C_i_/N.


$$\left(\overset{`}{\text{A}}=\text{arg}\,{\text{max}}_{\bar{\text{A}}}\,\left|{\overset{`}{\text{A}}}^{\text{T}}\,{\overset{`}{\text{S}}}_{\text{b}}\,\overset{`}{\text{A}}\right|/\left|{\overset{`}{\text{A}}}^{\text{T}}\,{\bar{\text{S}}}_{\text{b}}\,\bar{\text{A}}\right|+\left|{\overset{`}{\text{A}}}^{\text{T}}\,{\overset{`}{\text{S}}}_{\text{w}}\,\overset{`}{\text{A}}\right|\right)$$

$$when\left(\alpha =1,\gamma =\left(\text{tr}\left({\bar{\text{S}}}_{\text{i}}/N\right)+\text{M}\right)/M\right)$$

Classification using deep graph ConvoNet (convolutional network)- DG_ ConvoNet:

𝒢 = (𝒴, ℰ, ℋ) defines an undirected and connected graph, Here A and S are limited sets of | A| = S vertices as well as edges W ∈ ℝ^N × N^. Numerous variables in each vertex represent the graph signals. ℒ = D−W, where $\text{D}=\text{diag}\,\left({\overset{`}{d}}_{0},\cdots ,d_{N-1}\right)$ is a grading matrix designed in steps d_i_ = Σ_j_𝒲_i,j_ of vertex i. ${\left\{\chi_{\text{l}}\right\}}_{/,=0}^{N-1}$, as well as nonnegative eigenvalues 0 ≤ λ_0_ ≤ ⋯ λ_N−1_⋅ℒ. L is verified by a matrix of eigenvectors 𝒳 = [χ_0_,⋯,χ_N−1_] such that ℒ = 𝒳Λ𝒳^T^ where ℒ is a diagonal matrix of eigenvalues.

Instead of complex exponentials, the eigenvectors, ${\left\{\chi_{,}\right\}}_{/,=0}^{N-1}$ of Laplacian matrix L that meet perpendicularity criteria are utilized as breakdown bases for graph-structured data is defined as Eq. (12):


(12) $$\hat{\text{f}}\,\left(\lambda_{{}^{\prime }}\right)=\Sigma_{\text{n}=0}^{\text{N}-1}\,\chi_{,}^{\text{T}}\,\left(\text{n}\right)\,\text{f}\,\left(\text{n}\right)={𝒳}^{\text{T}}\,\text{f}$$

Inverse Fourier transformation is shown in Eq. (12):


(13) $$\text{f}\,\left(\text{n}\right)=\Sigma_{\text{l}=0}^{\text{N}-1}\,\hat{\text{f}}\,\left(\lambda_{{}^{\prime }}\right)\,\chi_{{}^{\prime }}\,\left(\text{n}\right)=x\,\hat{\text{f}}$$

In the Fourier domain, convolution is converted to a point-wise product, which can then be reconverted to vertex domain utilizing graph Fourier transform as well as convolution theorem, as shown in Eq. (14):


$$\text{f}*\text{g}=\Sigma_{/,=0}^{\text{N}-1}\,\hat{\text{f}}\,\left(\lambda_{/}\right)\,\overset{`}{\text{g}}\,\left(\lambda_{\zeta }\right)\,\chi_{⋏}\,\left(\text{n}\right)=𝒳\,\left(\left({𝒳}^{\text{T}}\,\text{f}\right)\cdot \left({𝒳}^{\text{T}}\,\text{g}\right)\right)$$

(14) $$=𝒳\,d\,ı\,a\,g\,\left(\overset{`}{\text{g}}\,\left(\lambda_{0}\right),\cdots ,\overset{`}{\text{g}}\,\left(\lambda_{\text{N}-1}\right)\right)\,{𝒳}^{\text{T}}\,\text{f}$$

**FIGURE 3:**
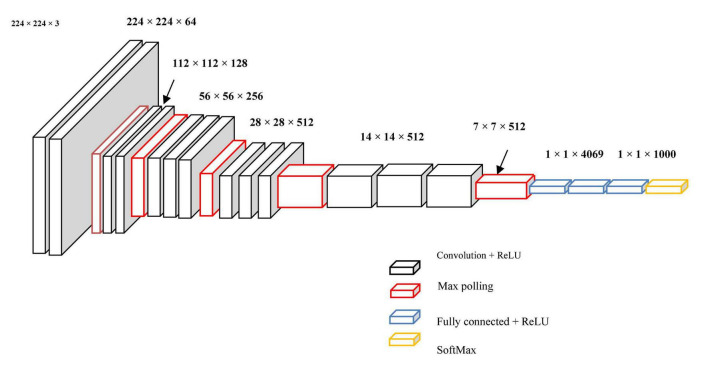
Graphical illustration of convolution f (n) and g (n).


The graph convolution process of 2 graph signals f(n) and g(n) is shown in Figure 3, and its transform, g () l, is called a Conv kernel. A set of free parameters θ_N−1_ in Fourier domain, i.e., Laplacian eigenspace is used to build this kernel. It can also be thought of as a function of eigenvalues, written as g(A). Convolution is then written as Eq. (15):

(15) $$\text{f}*\text{g}=x\,d\,ı\,a\,g\,\left(\theta_{0},\cdots ,\theta_{N-1}\right)\,{𝒳}^{T}\,\text{f}=𝒳\,𝒢\,\left(\Lambda \right)\,{𝒳}^{T}\,\text{f}$$

The convolution mentioned above on a graph has two drawbacks: (1) Each process involves an Eigen decomposition, which incurs high computational costs; (2) after this operation, the variable value of a vertex is associated with global vertices without considering its locality in space, which is inconsistent with CNNs’ local connections.

suggested a low-order polynomial approximation based on rapid localized convolution that depicts g(A) as a polynomial function of eigenvalues Eq. (16):


(16) $$𝒢\,\left(\Lambda \right)=\Sigma_{k=0}^{K}\,\theta_{k}\,\Lambda^{k}$$

θ_k_ is the polynomial order, and _k is a vector of polynomial coefficients. The convolution is then rewritten where K is a small positive integer, such as Eq. (17).


$$\text{f}*\text{g}=𝒳\,\left(\Sigma_{\text{k}=0}^{\text{K}}\,\theta_{\text{k}}\,{\text{Λ}}^{\text{k}}\right)\,{𝒳}^{\text{T}}\,\text{f}=\left(\Sigma_{\text{k}=0}^{\text{K}}\,\theta_{\text{k}}\,\left(𝒳\,{\text{Λ}}^{\text{k}}\,{𝒳}^{\text{T}}\right)\right)\,\text{f}$$

(17) $$=\Sigma_{\text{k}=0}^{\text{K}}\,\theta_{\text{k}}\,{ℒ}^{\text{k}}\,\text{f}$$

The convolution is performed by K multiplications of sparse matrix L, which speeds up computation by avoiding the Eigen decomposition procedure.

Update equation for a layer l is defined as Eq. (18):


$${\overset{`}{\text{h}}}_{\text{i}}^{\text{l}+1}={\text{O}}_{\text{h}}^{\text{l}}\,{\text{H}}_{\text{k}=1}\,\left(\Sigma_{\text{j}\in {\text{N}}_{\text{i}}}\,{\text{w}}_{\mathrm{ij}}^{\text{k},\text{l}}\,{\text{V}}^{\text{k},\text{l}}\,{\text{h}}_{\text{j}}^{\text{l}}\right)\,{\overset{`}{\text{e}}}_{\text{i}}^{\text{l}+1}={\text{O}}_{\text{e}}^{\text{l}}\,{\text{H}}_{\text{k}=1}$$

$$\left({\overset{`}{\text{w}}}_{\text{i},\text{j}}^{\text{k},\text{l}}\right)\,{\text{w}}_{\mathrm{ij}}^{\text{k},\text{l}}={\mathrm{softmax}}_{\text{j}}\,\left({\overset{`}{\text{w}}}_{\text{i},\text{j}}^{\text{k},\text{l}}\right)$$

(18) $$\text{w}\,{\overset{`}{\text{v}}}_{\text{i},\text{j}}^{\text{k},\text{l}}=\left(\frac{{\text{Q}}^{\text{k},\text{l}}\,{\text{h}}_{\text{i}}^{\text{l}}\cdot {\text{K}}^{\text{k},\text{l}}\,{\text{h}}_{\text{j}}^{\text{l}}}{\sqrt{{\text{d}}_{\text{k}}}}\right)\,{\text{E}}^{\text{k},\text{l}}\,{\text{e}}_{\text{i},{\text{j}}^{{}^{\prime }}}^{\text{l}}$$

with ${\text{Q}}^{\text{k,l}}{\text{, K}}^{\text{k,l}}{\text{, V}}^{\text{k,l}}{\text{, E}}^{\text{k,l}}\in {\text{Rd}}_{\text{k}},{\text{ O}}_{{\text{h}}^{{}^{\prime }}}^{\text{l}},{\text{ O}}_{\text{e}}^{\text{l}}\in {\text{R}}^{\text{d × d}},\text{ k}\in \{1,2,\text{…},\text{H}\}$ represents the number of attention heads, and where ${\text{O}}_{\text{h}}^{\text{l}}\in {\text{R}}^{\text{d }\times \text{ d}},{\text{V}}^{\text{k,l}}\in {\text{R}}^{{\text{d}}_{\text{k}}\;\times \text{ d}}\text{H}$ indicates the number of heads, L number of layers, d is the hidden dimension and d_k_ is the dimension of a head d H = dk. Note that h*^l^*_*i*_ is ith node’s feature at lth layer Eq. (19).


(19) $$\mathrm{cut}\,\left({\text{S}}_{\text{k}},{\overset{`}{\text{S}}}_{\text{k}}\right)=\sum_{{\text{v}}_{\text{i}}\in {\text{S}}_{\text{k}},{\text{v}}_{\text{j}}\in {\text{S}}_{\text{j}}}\text{e}\,\left({\text{v}}_{\text{i}},{\text{v}}_{\text{j}}\right)$$

where S_k_ is the k_*th*_ set of a given eigenvector, ${\overset{`}{\text{S}}}_{\text{k}}$ indicates residual sets excluding S_k_ and e(v_i_, v_j_) is an edge among vertex v_i_ and v_j_. The cut problem can be rewritten as follows when referring to several sets Eq. (20):


(20) $$\mathrm{cut}\,\left({\text{S}}_{1},{\text{S}}_{2},{\text{S}}_{3}\,\ldots \,{\text{S}}_{\text{g}}\right)=\frac{1}{2}\,\sum_{\text{i}=\text{k}}^{\text{g}}\mathrm{cut}\,\left({\text{S}}_{\text{k}},{\text{S}}_{\text{k}}\right)$$

The minimum cut problem is extensively researched in literature, with normalized cut reflecting a separate direction Eq. (21):


(21) $$\mathrm{Ncut}\,\left({\text{S}}_{1},{\text{S}}_{2}\,\ldots \,{\text{S}}_{\text{g}}\right)=\sum_{\text{k}=1}^{\text{g}}\frac{\mathrm{cut}\,\left({\text{S}}_{\text{k}},{\overset{`}{\text{S}}}_{\text{k}}\right)}{\text{vol}\,\left({\text{S}}_{\text{k}},\text{V}\right)}$$

wherever vol(S_k_, V) = Σ_v_i_∈S_k_,v_i_∈V^e^_ (v_i_, v_j_) is the entire grade of bulges from S_k_ in diagram g.

utilizing DL optimization to turn the minimum cut issue into a DL format Eq. (22):


(22) $${\text{L}}_{\mathrm{cut}}=\sum_{\mathrm{lower}\,\mathrm{sum}}\left[\left(\text{Y}⊘\Gamma \right)\,{\left(1-\text{Y}\right)}^{T}\right]\,⊙\text{A}+\sum_{\mathrm{lower}\,\mathrm{sum}}{\left(1^{T}\,\text{Y}-\frac{\text{n}}{\text{g}}\right)}^{2}$$

The normalized cut is the first term, and Y is defined as an n * g dimension matrix that indicates the neural network’s output. Finally, Γ, Y calculates A, which is the adjacency matrix Eq. (23).


(23) $${\text{H}}_{j}^{\left[l+1\right]}=\sigma \,\left(\sum_{i=1}^{F_{i\,n}}\left(\sum_{k=0}^{K}\theta_{i,j_{k}}\,{ℒ}^{k}\,{\text{H}}_{i}^{\left[l\right]}\right)+{\text{b}}_{j}^{\left[l\right]}\right)$$

**FIGURE 4:**
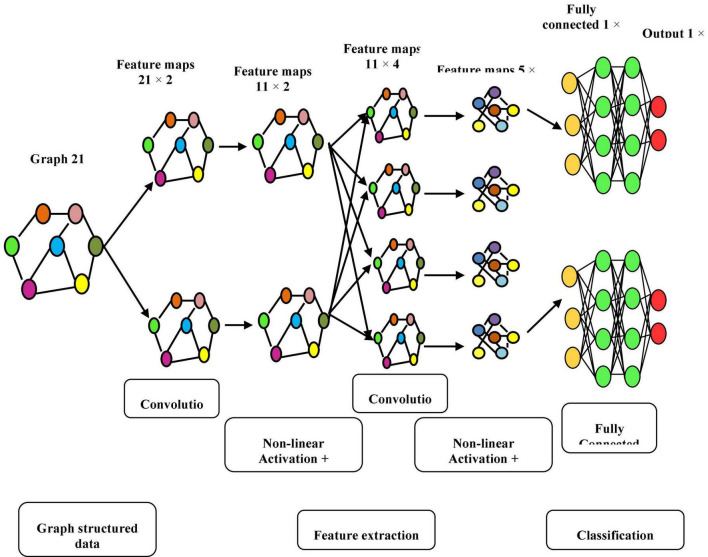
Model’s architecture with two convolutional layers.

Manifold convolutional and pooling layers, as well as one fully associated layer, make up the model. Figure 4 depicts the model’s architecture with two convolutional layers.

**Convolutional network:** Convolutional layers are the foundation of a convolutional neural network. It has some filters (or kernels) whose settings will be figured out as the training progresses. Typically, its filter’s size will be less than that of the image it’s applied. Each filter performs a convolution on the image, yielding an activation map. For convolution, the filtration is moved throughout the height & width of the image, and at each point in space, the dot product between each component of the filter & the input is measured. The implemented design with the Deep Graph CNN model can provide better heart disease prediction compared to earlier models. The main features of this design are to give less ToC and accurate diagnosis results compared to earlier models. Heart diseases had been predicted at the classification stage using the GS-CNN process. The shape-based features are more helpful to find the information medical image such that getting differentiation with training data.

## Performance analysis

A thorough experimental analysis was used to calculate the suggested hybrid technique performance. The proposed hybrid technique was tested on a PC with the following parameters: Intel(R) Core (TM) i5-7500 CPU, 32-bit Windows 7 OS, 4 GB RAM with SciPy, NumPy, Pandas, Keras and Matplotlib frameworks and Python 2.7 as the programming language.

### Dataset description

Public Health Dataset, which dates from 1988 and consists of four databases: Cleveland, Hungary, Switzerland and Long Beach V, was used for this study. Even though there are 76 qualities in total, including expected attributes, all published studies only utilize a selection of 14 of them.

### Information on heart disease

The clinical HD data used in this study came from 303 patients at CCF in Cleveland, Ohio, in the US. Dataset was collected from UCI_MLRepository (Hinton and Salakhutdinov, 2006), part of the Heart Disease Database. There were 75 attributes and a target attribute in each of the 303 clinical situations. The target attribute was an integer ranging from 0 to 4, indicating whether a patient had HD [0] or not [1, 2, 3]. Target qualities for the absence or presence of cardiac disease in patients were ascribed to binary values of 0 and 1 for this study. There were 125 cases with heart disease (44.33%) and 157 cases without heart disease (55.67%) among the 282 total clinical episodes. A total of 76 raw attributes were used to describe each clinical event. Due to missing values among other raw variables, only 29 of the raw attributes were used in the building of DNN models (Djenouri et al., 2022; Mezair et al., 2022).

**FIGURE 5:**
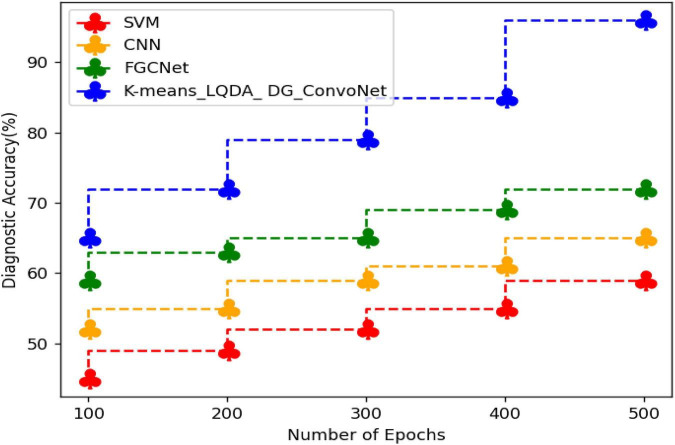
Comparative analysis of diagnostic accuracy.

Table 2 and Figure 5 show comparative analysis in diagnostic accuracy for proposed K-means_LQDA_ DG_ConvoNet. The diagnostic accuracy has been analyzed based on the number of epochs the neural network carries out. The epochs are taken as 100, 200, 300, 400 and 500. For all the iterations of the neural network, the proposed K-means_LQDA_ DG_ConvoNet obtained optimal results than the existing technique. The accuracy obtained in the diagnosis of disease by proposed K-means_LQDA_ DG_ConvoNet is 96% and existing SVM achieved 59% for 500 epochs and CNN obtained 65%, FGCNet attained 72%.

**TABLE 2:** Comparative analysis of diagnostic accuracy.

Number of epochs	SVM	CNN	FGCNet	K-means_LQDA_DG_ConvoNet
100	45	52	59	65
200	49	55	63	72
300	52	59	65	79
400	55	61	69	85
500	59	65	72	96

**FIGURE 6:**
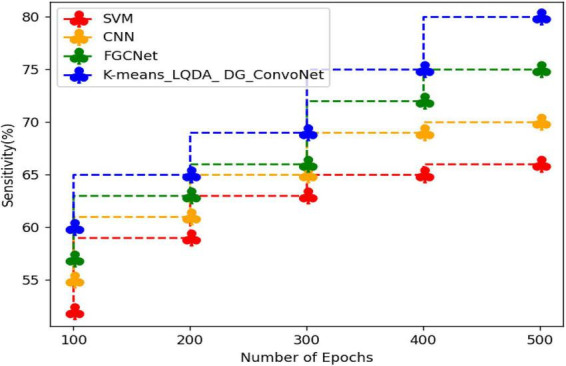
Comparative analysis of sensitivity.

Table 3 and Figure 6 show comparative sensitivity analysis for proposed K-means_LQDA_ DG_ConvoNet. The sensitivity calculation refers prediction of the true positive and false positive rate of the proposed technique in diagnosing heart disease. The sensitivity obtained in disease diagnosis by proposed K-means_LQDA_ DG_ConvoNet is 80% for 500 epochs and existing SVM achieved 66% for 500 epochs and CNN obtained 70%, FGCNet attained 75%.

**TABLE 3:** Comparative analysis of sensitivity.

Number of epochs	SVM	CNN	FGCNet	K-means_LQDA_DG_ConvoNet
100	52	55	57	60
200	59	61	63	65
300	63	65	66	69
400	65	69	72	75
500	66	70	75	80

**FIGURE 7:**
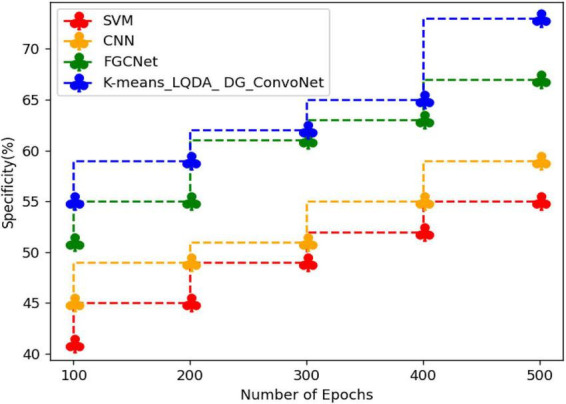
Comparative analysis of specificity.

Table 4 and Figure 7 show comparative analysis in terms of specificity for proposed K-means_LQDA_ DG_ConvoNet. The specificity calculation relates to the percentage of real negatives projected as negatives. This means that a part of true negatives is forecasted as positives, which is denoted as false positives in the suggested method for identifying HD. The specificity obtained in the diagnosis of disease by proposed K-means_LQDA_ DG_ConvoNet is 73% for 500 epochs and existing SVM achieved 55% for 500 epochs and CNN obtained 57%, FGCNet attained 67%.

**TABLE 4:** Comparative analysis of specificity.

Number of epochs	SVM	CNN	FGCNet	K-means_LQDA_DG_ConvoNet
100	41	45	51	55
200	45	49	55	59
300	49	51	61	62
400	52	55	63	65
500	55	59	67	73

**FIGURE 8:**
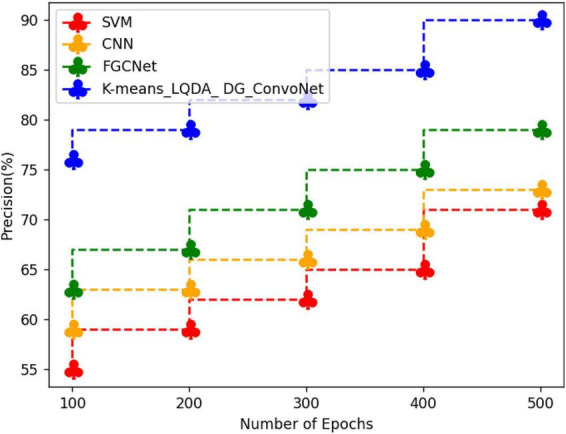
Precision analysis differentiation.

Table 5 and Figure 8 show qualified examination in terms of Precision for proposed K-means_LQDA_ DG_ConvoNet. The precision calculation mentions the number of true positives separated by the whole number of positive calculations made by the suggested technique in diagnosing heart disease, as well as the superiority of a positive forecast made by the proposed technique. The precision obtained in the diagnosis of disease by proposed K-means_LQDA_ DG_ConvoNet is 90% for 500 epochs and existing SVM achieved 71% for 500 epochs and CNN obtained 73%, FGCNet attained 79%.

**TABLE 5:** Comparative analysis of precision.

Number of epochs	SVM	CNN	FGCNet	K-means_LQDA_DG_ConvoNet
100	55	59	63	76
200	59	63	67	79
300	62	66	71	82
400	65	69	75	85
500	71	73	79	90

**FIGURE 9:**
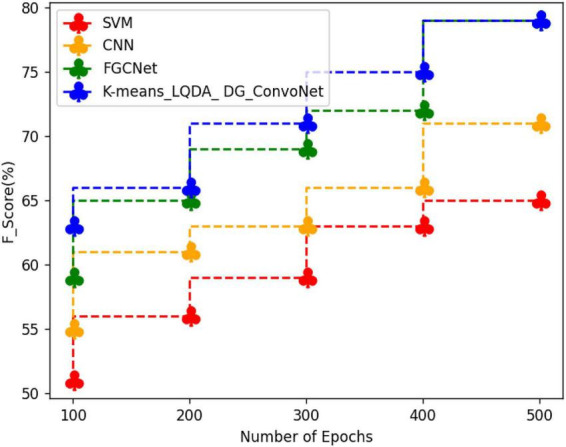
Comparative analysis of F-Score.

Table 6 and Figure 9 show a comparative analysis in terms of F-Score for proposed K-means_LQDA_ DG_ConvoNet. The F-Score computation is utilized to assess binary classification techniques which categorize examples as “positive” or “negative.” *F*-score is shown as the harmonic mean of precision and recall. For example, *F*-Score obtained in the diagnosis of disease by proposed K-means_LQDA_ DG_ConvoNet is 79% for 500 epochs and existing SVM achieved 65% for 500 epochs and CNN obtained 71%, FGCNet attained 79%.

**TABLE 6:** Comparative analysis of *F*-Score.

Number of epochs	SVM	CNN	FGCNet	K-means_LQDA_DG_ConvoNet
100	51	55	59	63
200	56	61	65	66
300	59	63	69	71
400	63	66	72	75
500	65	71	79	79

**FIGURE 10:**
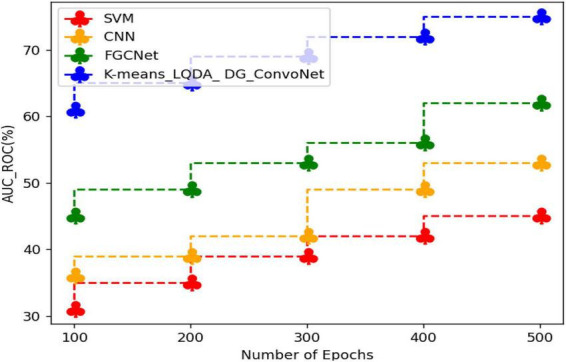
ROC curve analysis.

Table 7 and Figure 10 show an examination of the area under the ROC curve for proposed K-means_LQDA_ DG_ConvoNet. The calculation of the extent under the ROC curve is a measure of a classifier’s ability to distinguish between classes as well as used as an instant of the ROC curve.

**TABLE 7:** ROC curve on various methods.

Number of epochs	SVM	CNN	FGCNet	K-means_LQDA_DG_ConvoNet
100	31	36	45	61
200	35	39	49	65
300	39	42	53	69
400	42	49	56	72
500	45	53	62	75

**FIGURE 11:**
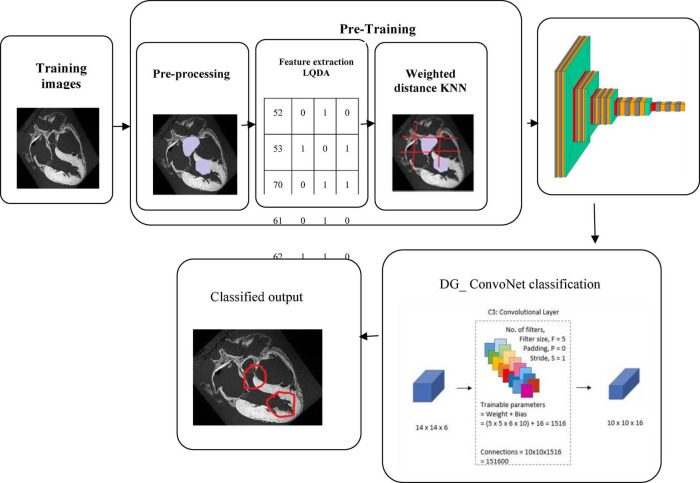
Classification of heart disease prediction.

AUC indicates how well the method differentiates between positive and negative classes. F-Score obtained in the diagnosis of disease by proposed K-means_LQDA_ DG_ConvoNet is 75% for 500 epochs and existing SVM achieved 45% for 500 epochs and CNN obtained 53%, FGCNet attained 62% shown in Figure 11.

## Conclusion

The proposed work is a novel technique for detecting heart disease based on IoT sensor data with a monitoring application using deep learning architectures. Here, the input data has been collected from IoT sensor data from the University of California Irvine machine learning repository. The collected data has been processed for noise removal and clustered based on K-means clustering. The clustered data has been extracted using Linear Quadratic Discriminant Analysis where the features of clustered data have been extracted. The extracted features have been classified using the deep graph ConvoNet (convolutional network)- DG_ConvoNet. The diagnostic accuracy of 96%, sensitivity of 80%, specificity of 73%, precision of 90%, *F*-Score of 79%, and area under the ROC curve of 75% are obtained by the proposed classification and prediction model, according to the testing findings. Our strong results clearly show the strength of our methodology and DG_ConvoNet. In the future, we wish to test our system model on other datasets and also look at implementing the DG_ConvoNet for other diseases.

## Data availability statement

Publicly available datasets were analyzed in this study. This data can be found here at doi: 10.1136/bmjopen-2020-044070.

## Author contributions

KS and VR contributed to the conception and design of the study. GS performed the statistical analysis. KS and JL wrote the first draft of the manuscript. All authors contributed to the manuscript revision, read, and approved the submitted version.
